# Host Plant Species of *Bemisia tabaci* Affect Orientational Behavior of the Ladybeetle *Serangium japonicum* and Their Implication for the Biological Control Strategy of Whiteflies

**DOI:** 10.3390/insects11070434

**Published:** 2020-07-11

**Authors:** Mi Tian, Lili Xu, Jun Jiang, Shize Zhang, Tongxian Liu, Yongyu Xu

**Affiliations:** 1 State Key Laboratory of Crop Stress Biology for Arid Areas, Northwest A&F University, Yangling 712100, China; mitian1993@163.com (M.T.); xu-lili@nwsuaf.edu.cn (L.X.); junjiang@nwsuaf.edu.cn (J.J.); txliu@nwsuaf.edu.cn (T.L.); 2 College of Plant Protection, Shandong Agricultural University, Taian 271018, China; xuyy@sdau.edu.cn

**Keywords:** *Serangium japonicum*, *Bemisia tabaci*, orientational behavior, host plant species, plant volatiles, biological control

## Abstract

*Serangium japonicum* Chapin (Coleoptera: Coccinellidae) is a predominant predator with a preference for *Bemisia tabaci* (Gennadius) (Hemiptera: Aleyrodidae). To date, the orientational behavior of *S. japonicum* toward *B. tabaci*-infested plants has seldom been reported. In this study, greenhouse cage experiments and bioassays with wind tunnels, a Y-tube olfactometer and *B. tabaci*-induced plant volatiles were executed to clarify this behavior. In greenhouse cage experiments, *B. tabaci* adults significantly preferred eggplant, cucumber and tobacco to cotton and tomato, whereas *S. japonicum* adults preferred *B. tabaci*-infested eggplant, cucumber and cotton to tobacco and tomato. In wind tunnel bioassays, *B. tabaci* showed a clear preference for eggplant, cucumber and tobacco. Compared with *B. tabaci*-infested eggplant, cucumber or cotton, *B. tabaci*-infested tobacco was rarely visited by *S. japonicum*. In Y-tube bioassays, *S. japonicum* did not distinguish between *B. tabaci*-infested and uninfested eggplant. Nine common plant volatiles were detected in different plant species, suggesting that these volatiles may play an important role in the process by which *S. japonicum* looks for prey. In light of the current results, we discuss the implications of our findings and put forward to a new strategy—i.e., an eggplant + *B. tabaci* + *S. japonicum* system—to control *B. tabaci* damage in the integrated management of whitefly.

## 1. Introduction

An agroecosystem includes the food web and infochemical web, in which pests and predators utilize chemical information to search for food resources [1]. Host plants play a bridging role among the plant‒prey–natural enemy tritrophic system. Plants provide habitat, food and refuge for pests and their natural enemies, and they also transfer basic communication information between the pests and natural enemies [2]. Plants are frequently attacked by all kinds of herbivores, including chewing–feeding and sap-feeding pests, and the pests not only result in physical wounds but also trigger underlying plant defense responses. Injured plants can release volatiles, known as herbivore-induced plant volatiles (HIPVs), to attract predators and parasitoids [3]. The primary functions and mechanisms of HIPVs in tritrophic interactions have been reviewed in detail [4].

Abundant evidence has proved that HIPVs play an important role in parasitoids locating hosts, and increasing amounts of data have also confirmed that HIPVs can attract predators. Aphid-induced plant volatiles were found to attract the ladybeetles *Harmonia axyridis* (Pallas) (Coleoptera: Coccinellidae), *Coccinella septempunctata* (L.) (Coleoptera: Coccinellidae) and *Propylaea japonica* (Thunberg) (Coleoptera: Coccinellidae) [5,6,7]; the emission of volatiles by citrus trees to repel *P. japonica* when infested simultaneously by *Diaphorina citri* Kuwayama (Hemiptera: Psyllidae) and Citrus Huanglongbing [8] was also detected. However, little is known about how whitefly-infested plants affect the performance of predatory ladybeetles [9]. 

The cotton whitefly *Bemisia tabaci* (Gennadius) Middle East-Asia Minor 1 (MEAM1, commonly known as B biotype) (Hemiptera: Aleyrodidae) is a major pest of many agronomic and horticultural crops, infesting more than 600 host plant species and causing great economic losses to agricultural production [10]. *Bemisia tabaci* has a high resistance to most pesticides, which causes concerns regarding the excessive use of insecticides. Therefore, biological control has been considered as a safe and sustainable way to alleviate the damage of *B. tabaci.* The biological control agents of *B. tabaci* include parasitoids, predatory ladybeetles, pathogenic microorganisms and so on [11,12,13]. The ladybeetle *Serangium japonicum* Chapin (Coleoptera: Coccinellidae) is a strong predator of whiteflies, and the adults have a lengthy longevity and high fecundity [12,14,15]; thus, the ladybeetle has drawn much attention regarding the control of *B. tabaci* [12,16,17,18].

Plants grow in complex multi-species communities, so natural enemies are always confronted with a mixture of plant odors. We hypothesized that different host plant species of *B. tabaci* might affect the orientational choice of *S. japonicum* as the ladybeetle locates its prey by using volatiles from their prey or prey-infested host plants, which may affect the biological control efficiency of *S. japonicum* against *B. tabaci*. Therefore, we studied (1) the preferences of *B. tabaci* and its natural enemy *S. japonicum* adults for different plants in greenhouse cage experiments, (2) the responses of *B. tabaci* and *S. japonicum* adults toward different host plants in a wind tunnel, (3) the performance of *S. japonicum* in a Y-tube olfactometer and (4) the volatiles collected from plants. The present work provides valuable information for the maximization of the biological control efficiency when utilizing *S. japonicum* to control *B. tabaci* in different plant species. In view of the current results, we also propose a new strategy—i.e., an eggplant + *B. tabaci* + *S. japonicum* system—to control *B. tabaci* damage in the integrated management of whitefly.

## 2. Materials and Methods

### 2.1. Plant Cultivation and Insect Breeding

Five different plant species, namely eggplant (*Solanum melongena* L. var. Xinyou zilong changqie), cucumber (*Cucumis sativus* L. var. Jinchun sihao), cotton (*Gossypium hirsutum* L. var. Luzao), tobacco (*Nicotiana tabacum* L. var. Qinyan 95) and tomato (*Lycopersicon esculentum* Mill var. Zhongshu sihao), were obtained from Yangling Nongcheng Seed Supplement Company (Yangling, Shaanxi, China). Each seed was grown separately in a potting tray containing a mixture of commercial peat mosses (60%) (Pindstrup Mosebrug A/S, Ryomgaard, Denmark), perlite (20%) and vermiculite (20%). The plants were grown inside a climate room, in which environmental conditions were maintained at 25 ± 1 °C, with 65 ± 5% RH and a 14/10 h (light/dark, L/D) photoperiod. 

*Bemisia tabaci* and *S. japonicum* adults were collected by aspiration from tomato and eggplant plants, respectively, on an experimental farm of Northwest A&F University. Whitefly adults were identified as *B. tabaci* MEAM1 through mitochondrial cytochrome oxidase I (mtCOI) gene amplification. Potted cabbage plants (*Brassica oleracea* L., Qingan 70) were used for maintaining *B. tabaci*, and potted cabbage with mixed ages of *B. tabaci* was used to rear *S. japonicum*. All insect colonies were kept in nylon gauze cages (60 × 60 × 60 cm) in an insectary at 20–30 °C with 65 ± 5% RH and a 14/10 h L/D photoperiod.

Cabbage leaves containing fourth instar nymphs of *B. tabaci* were picked and placed in small Petri dishes (3 cm in diameter, 1 cm in height) to obtain newly emerged adults for subsequent tests. Newborn *S. japonicum* larval individuals were raised in the same-sized Petri dishes with an agar layer. Clear Petri dishes were replaced, and sufficient food was provided daily until adult emergence. Petri dishes were maintained in an incubator (25 ± 1 °C, 65 ± 5% RH, 14/10 h L/D).

### 2.2. Preparation of B. tabaci-Infested Plants

(a) For greenhouse experiments, two weeks after germination, seedlings were singly transplanted into plastic pots (15 × 10 cm) filled with the same substrate and plants were fertilized once every week for five weeks. The composite fertilizer contained N (15%), P_2_O_5_ (20%), and K_2_O (20%), which was produced by Mei Sheng’s Chemical Fertilizer Co., Ltd., Yan Tan, China. Of the five plant species, seven weeks after seeding, each plant was separately placed into a nylon cage (30 × 30 × 60 cm) and exposed to injury from 25 pairs of *B. tabaci* adults for seven days. The plants were used for subsequent testing after whitefly adults were removed.

(b) For wind tunnel/Y-tube olfactometer/gas chromatography–mass spectrometry (GC–MS) extract volatile bioassays, plants that had germinated for seven weeks were unable to fit into odor source bottles, so the growth duration of plants had to be shortened to five weeks after germination. Two weeks after germination, the plants were individually transplanted into plastic pots (10 × 8 cm) containing the same mixture, and water and fertilizer management were provided as described above. After three weeks, these plants were individually placed in 5 L polyethylene oil drums with nylon gauze on both sides in the incubator (25 ± 1 °C, 65 ± 5% RH, 14/10 h L/D). Fifty pairs of newly emerged *B. tabaci* adults were introduced into each oil drum to infest for seven days. Finally, infested plants were used in experiments after adults were removed. 

### 2.3. Greenhouse Cage Experiments

Multiple-choice experiments were executed to evaluate the preference of *B. tabaci* adults for five plant species and the preference of *S. japonicum* adults for five *B. tabaci*-infested plant species.

#### 2.3.1. Multi-Choice Experiments of *B. tabaci* Adults

Each replicate cage had five uninfested plant species (eggplant, cucumber, tobacco, cotton and tomato), which were uniformly placed in a circle in a nylon mesh cage (60 × 60 × 60 cm). An upended plastic pot (15 × 10 cm) with a Petri dish (9 cm in diameter, 1 cm in height) was put in the center of the cage to act as an insect release platform. Twenty-five pairs of newly emerged *B. tabaci* adults (<24 h) were released into the platform for ad libitum feeding. The number of *B. tabaci* adults on each plant was counted at 24, 72 and 120 h after release. Finally, two pieces of leaves from each plant were collected 120 h after the whiteflies were released, and then the number of *B. tabaci* eggs was checked by a stereoscopic microscope in one randomly selected square centimeter. The position of each plant species in the cage changed randomly during each replication. The experiments were repeated six times.

#### 2.3.2. Multi-Choice Experiments of *S. japonicum* Adults to *B. tabaci*-Infested Plant Species

Five *B. tabaci*-infested plant species were placed in a nylon mesh cage (60 × 60 × 60 cm). The spatial treatment was arranged as described in Section 2.3.1, and 20 virgin adults of *S. japonicum* (newborn within a week) with 24 h of starvation were put into the release platform for ad libitum feeding. The number of *S. japonicum* adults on each plant (all leaves) was recorded at 1, 3, 6, 24, 72 and 120 h after the ladybeetle release. This experiment was repeated five times simultaneously.

### 2.4. Wind Tunnel Bioassays

On the basis of the results of the greenhouse experiment, the responses of *B. tabaci* and *S. japonicum* adults toward different host plants were further investigated in a wind tunnel. The wind tunnel was installed following the procedure of Feng et al. [19]. The device was cleaned with 70% alcohol and given 2 h of idle time before each experiment. An upended plastic pot (10 × 8 cm) with a Petri dish (9 cm in diameter, 1 cm in height) was applied as a release platform. Each wind tunnel device contained two different potted plants. Each plant was placed 5 cm from the edge of the air inlet, the insect release platform was placed 5 cm from the edge of the air outlet, and the two potted plants were 15 cm from the wind tunnel. Plants were left to stabilize for 30 min before the beginning of the experiment, and the positions of the plants were randomized per repetition.

#### 2.4.1. Two-Choice Tests of *B. tabaci* Adults

Five different plant species were divided into preferential and non-preferential plants of *B. tabaci* adults according to the results of greenhouse cage experiments. The preferential plants were eggplant, cucumber and tobacco, and non-preferential plants were cotton and tomato. Uninfested plant odor sources included six different groups: (1) cotton *versus* eggplant, (2) cotton *versus* cucumber, (3) cotton *versus* tobacco, (4) tomato *versus* eggplant, (5) tomato *versus* cucumber and (6) tomato *versus* tobacco. Fifty pairs of newly emerged *B. tabaci* adults were put on the release platform for ad libitum choice. Thereafter, the numbers of *B. tabaci* adults on each plant were counted after 24 h. Each group was tested in four identical devices simultaneously. 

#### 2.4.2. Two-Choice Tests of *S. japonicum* Adults 

Five different whitefly-infested plants were classified as preferential and non-preferential plants for *S. japonicum* adults on the basis of the data from greenhouse cage experiments. The preferential plants included *B. tabaci-*infested eggplant, cucumber and cotton, and the non-preferential plants involved *B. tabaci-*infested tobacco and tomato. Tomato was not considered in this experiment because it was not only the least visited by *B. tabaci* adults in greenhouse and wind tunnel experiments but also seldom located by *S. japonicum* adults in the greenhouse. Ten *S. japonicum* adults (newborn within a week) with 24 h of starvation were put on the release platform for random selection for each device. The total numbers of *S. japonicum* adults on each plant (all leaves) were recorded at 24 h after the initial release. Three groups of plant odor sources were tested: (1) *B. tabaci-*infested tobacco *versus B. tabaci-*infested eggplant, (2) *B. tabaci-*infested tobacco *versus B. tabaci-*infested cucumber and (3) *B. tabaci-*infested tobacco *versus B. tabaci-*infested cotton. At least 40 ladybeetle adults were used in each group.

### 2.5. Y-Tube Olfactometer Experiments

According to the results of greenhouse cage experiments and wind tunnel bioassays, the *B. tabaci*-infested plants—i.e., eggplant, cucumber and cotton—showed similar tendencies in terms of attracting *S. japonicum* adults. However, it was still unclear whether the attraction was caused by the volatiles of plants, the visual stimulation of the ladybird or a combination of the two. Accordingly, a Y-tube olfactometer was executed to evaluate whether the volatile compounds of these plant species had different effects on the performance of *S. japonicum*. Different odor source groups were tested: (1) uninfested eggplant *versus B. tabaci*-infested eggplant, (2) uninfested cucumber *versus B. tabaci*-infested cucumber, (3) uninfested cotton *versus B. tabaci*-infested cotton, (4) *B. tabaci*-infested cotton *versus B. tabaci*-infested eggplant, (5) *B. tabaci*-infested cotton *versus B. tabaci*-infested cucumber and (6) *B. tabaci*-infested eggplant *versus B. tabaci*-infested cucumber.

The Y-tube olfactometer device was installed according to Darshanee et al. [20]. Briefly, purified air generated from an air pump (Beijing BCHP Analytical Technology Institute, Beijing, China) was filtered by an activated charcoal jar and a distilled water jar, then passed through a rotor flowmeter set at a constant airflow of 200 mL/min, entered 3 L volatile source bottles and finally flowed through each branch of the Y-tube olfactometer. Each arm of the Y-tube olfactometer had a 2.5 cm internal diameter and 10 cm length, and the two side-arms were approximately 60°. A piece of gauze was used in the junction between the Y-tube olfactometer and a Teflon rubber tube to prevent the insect from entering the Teflon rubber tube. Potted plants were covered with oven bags (EasyOven polythene bags, Reynolds Kitchens, USA) and tied with Teflon tape to fence out volatiles from a pot with soil, and then they were placed into volatile source bottles. Plants were kept behind a black cloth to prevent insects from visually recognizing them during the experiment. Potted plants were put in 3 L volatile source bottles for 30 min to allow odors to stabilize before testing. All glass devices and Teflon rubber tubes were cleaned by 70% alcohol solution and deionized water. Glass devices were baked at 120 °C and tubes for at 70 °C each for 2 h.

The discrimination criterion of *S. japonicum* behavior was calculated to according to the definition of *Harmonia axyridis* [21]. The naïve ladybeetle (newborn within a week) was placed at the downwind end of the main tube. An effective choice was recorded when the individual chose and entered one arm tube, passed one-third of the length of the arm tube and remained for 30 s. If an individual did not make a decision within 5 min, it was considered a non-responsive insect. After every five ladybeetles were tested, the Y-tube was reoriented by 180° to reduce the error that could be introduced by the device position. After every 10 ladybeetles were assayed, the Y-tube was replaced with a new one to avoid odor bias from its kind. After every 20 ladybeetles were examined, the glass chambers, potted plants and Teflon tubes were replaced with new ones to avoid plant odor effects. Data were collected for 40 individual ladybeetles. The experiments were conducted between 14:00 and 18:00 on several consecutive days.

### 2.6. Volatile Compound Collection and Analysis

The dynamic headspace volatiles from eggplant, cucumber and cotton were collected and analyzed by a gas chromatography–mass spectrometry system (GC–MS). A solid-phase microextraction (SPME) fiber that was attached with polydimethylsiloxane–divinylbenzene (PDMS-DVB, 65 mm; Supelco, Bellefonte, PA, USA) was used to collect plant volatiles [20]. Devices were installed as described above but not connected to the Y-tube, and finally, the SPME fiber absorbed the odors from the upper exits of volatile source bottles. Prior to volatile collection, each plant was sealed for 45 min in volatile source bottles to increase the concentration of volatiles. The volatile collection had five repetitions for each odor source using a new plant sample. 

The SPME fiber was inserted in a gas chromatograph (GC) injection port at 250 °C for 30 min for activation. After 30 min, the SPME fiber was inserted into the volatile source bottles with plants to absorb dynamic headspace volatiles. At the same time, a filter paper (5 × 2 mm) with 25 µL hexane solution of heptyl acetate at 10 ng/µL was added as an internal standard. The collection of volatiles was maintained for 45 min according to preliminary results. After 45 min, the SPME needle was carefully taken out and immediately inserted into the GC–MS system, and desorption was carried out for 5 min. The GC–MS analysis of headspace extracts was carried out on a GC (TRACE 1310, Thermo Fisher Scientific, Waltham, MA, USA) connected with an MS (ISQ Single Quadrupole MS, Thermo Fisher Scientific) fitted with a DB-17 MS UI capillary column (30 m, 0.25 mm i.d., 0.25 μm film, Agilent). GC conditions were consistent with those in [20]. The initial GC oven temperature was set to 40 °C for 2 min, ramped up at 8 °C min^−1^ to 280 °C and held for 5 min. Helium flow was carried out at a rate of 1.0 mL/min, with a transfer line at 280 °C, ion source temperature of 280 °C, electron impact ionization energy of 70 eV, emission current of 25 µA and a scan range from 45 to 500 amu.

Individual volatile compounds were tentatively identified by comparing the fragment ions to the NIST 2011 (National Institute of Standards and Technology, Washington, DC, USA) mass spectral library with the Xcalibur program (Ver.2.1, Thermo Electron Corporation, San Jose, CA, USA). Part of the result depended on a retention index, which was calculated from a homologous series of n-alkane standards (C7–C40, Sigma, Louis, MO, USA) and then compared to those of components from the literature.

### 2.7. Data Analysis

The number of *B. tabaci* adults on each uninfested plant and the numbers of *S. japonicum* adults on each *B. tabaci*-infested plant were normalized first by using the square-root transformation $\sqrt{x+0.5}$. Then, an ANOVA followed by a Tukey’s HSD or Dunnett’s T3 test was used to analyze the data from greenhouse experiments and based on the homogeneity of variance, and an independent samples t-test was applied for the wind tunnel bioassay data to analyze the significance at *p* < 0.05. In Y-tube assays, the data from the choice of ladybeetle were first transformed into percentages and then analyzed by χ^2^ tests with one degree of freedom at *p* < 0.05, and the 50% probability of choice for each odor arm was assumed for each group. The original peak areas of plant volatiles were transformed according to Geiselhardt et al. [22]; i.e., Z_i,j_ = ln [Y_i,j_/g(Y_j_) + 0.01], where Z_i,j_ is the mean standardized area of peak i for plant j, Y_i,j_ is the standard for the area of peak i for plant j and g(Y_j_) is the geometric mean of all identified peak areas for plant j. The constant 0.01 was adopted to ensure that the transformation formula was appropriate for all plants. A MANOVA with PCA was applied to analyze the significance of each volatile component [23,24]. The experimental data were analyzed using the package IBM SPSS Statistics 20 (SPSS, Inc., Chicago, IL, USA).

## 3. Results

### 3.1. Greenhouse Cage Experiments

**Figure 1:**
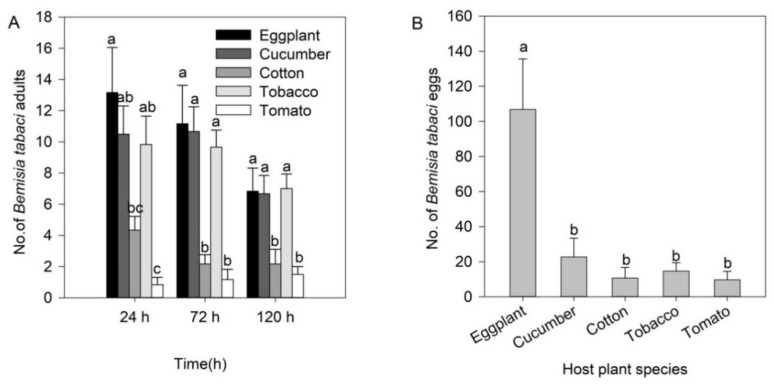
Numbers (mean ± SE) of *Bemisia tabaci* adults (**A**) and eggs after adults had infested plants for 120 h (**B**) for each plant in greenhouse cage experiments. Different lowercase letters mean significant differences among plant species at the same time at *p* < 0.05.

Greenhouse cage experiments showed that the numbers of *B. tabaci* adults and eggs were affected by different plant species (Figure 1). The maximum number of *B. tabaci* adults per plant was on eggplant at 24 h (F_(4, 25)_ = 11.763, *p* < 0.001), 72 h (F_(4, 25)_ = 13.190, *p* < 0.001) and 120 h (F_(4, 25)_ = 8.312, *p* < 0.001) (Figure 1A). Mean numbers of *B. tabaci* adults markedly varied from 13.2 to 6.8 per eggplant, 10.5–6.7 per cucumber, 9.8–7.0 per tobacco, 4.3–2.2 per cotton, and 0.8–1.5 per tomato from 24 to 120 h after adults were released, respectively (Figure 1A). After *B. tabaci* adults injured plants for 120 h, the number of eggs on eggplant was significantly higher than that on other plant species (F_(4, 25)_ = 8.564, *p* < 0.001; Figure 1B). 

**Figure 2:**
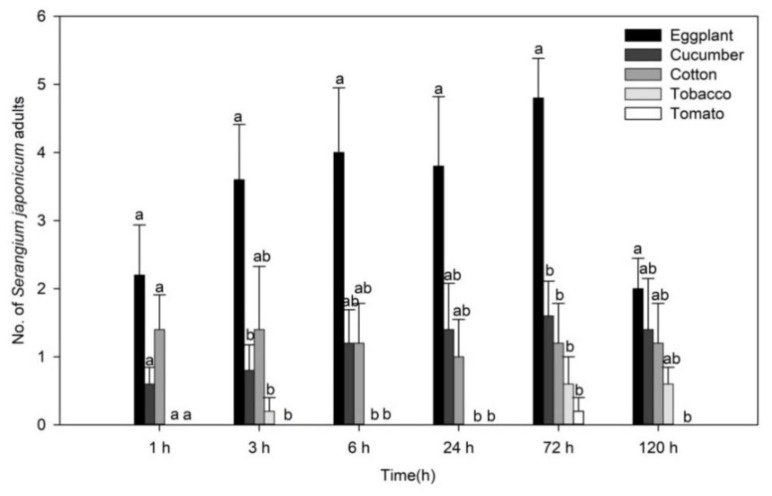
Numbers (mean ± SE) of *Serangium japonicum* adults on each plant in greenhouse cage experiments. Different lowercase letters mean significant differences among different plant species at the same time at *p* < 0.05.

The number of *S. japonicum* adults on each *B. tabaci*-infested plant greatly differed across plant species (Figure 2). More adults were found on eggplant, cucumber and cotton at each time interval than on other plant species (1 h: F_(4, 20)_ = 7.813, *p* = 0.001; 3 h: F_(4, 20)_ = 7.585, *p =* 0.001; 6 h: F_(4, 20)_ = 10.351, *p* < 0.001; 24 h: F_(4, 20)_ = 8.507, *p* < 0.001; 72 h: F_(4, 20)_ = 10.726, *p* < 0.001; and 120 h: F_(4, 20)_ = 3.020, *p =* 0.042; Figure 2). Moreover, *S. japonicum* adults clearly preferred eggplant during the investigation. In contrast, *S. japonicum* adults were only found on tomato at 72 h, and the number was lowest compared with other plant species. No *S. japonicum* adult was found on tobacco at 1 h, 6 h or 24 h after initial release (Figure 2). 

### 3.2. Wind Tunnel Bioassays

**Figure 3:**
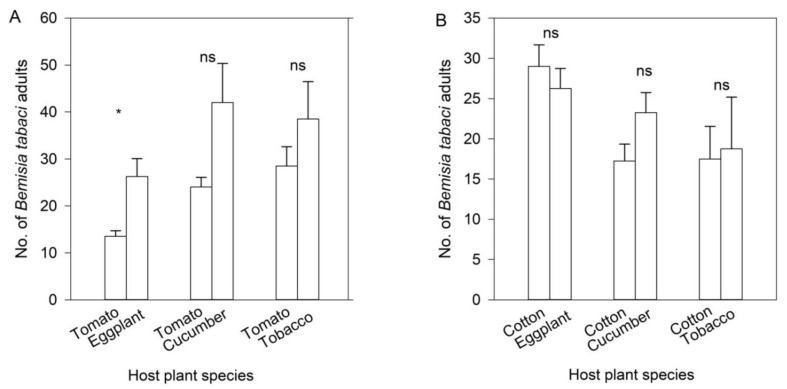
Numbers (mean ± SE) of *Bemisia tabaci* adults on each plant in the wind tunnel assays. (**A**) Non-preferential tomato *versus* preferential eggplant, cucumber and tobacco. (**B**) Non-preferential cotton *versus* preferential eggplant, cucumber and tobacco. The asterisk means significant difference and ns means no difference in each group at *p* < 0.05.

The number of *B. tabaci* adults on eggplant was significantly higher than that on tomato when they were simultaneously offered (t_6_ = −3.200, *p =* 0.019; Figure 3A). By contrast, *B. tabaci* adults did not show a significant preference between cucumber and tomato (t_6_ = −2.256, *p =* 0.065) or between tobacco and tomato (t_6_ = −1.119, *p =* 0.306), although the numbers of the whitefly appeared to have been risen on cucumber and tobacco (Figure 3A).

It was an unexpected phenomenon that *B. tabaci* adults were more concentrated on cotton than on eggplant, but the difference was not significant (t_6_ = 0.742, *p =* 0.486; Figure 3B). *Bemisia tabaci* adults did not show a significant preference between cotton and cucumber (t_6_ = −1.811, *p =* 0.120) or between cotton and tobacco (t_6_ = −0.020, *p =* 0.875; Figure 3B). 

**Figure 4:**
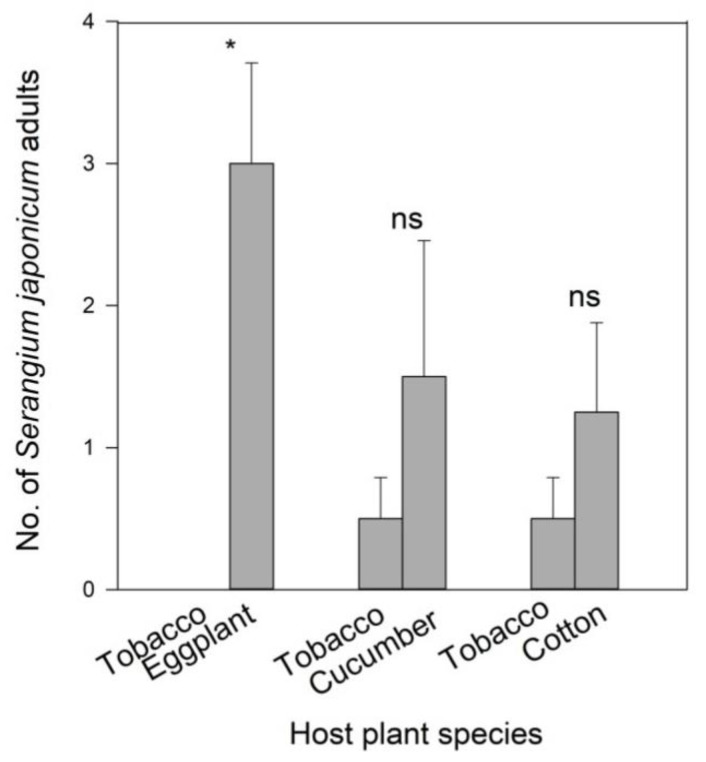
Numbers (mean ± SE) of *Serangium japonicum* adults on each plant in wind tunnel assays. The asterisk means a significant difference and ns means no difference in each group at *p* < 0.05.

When *B. tabaci*-infested eggplant and tobacco were offered simultaneously, *S. japonicum* adults markedly preferred *B. tabaci*-infested eggplant to tobacco (t_6_ = −6.309, *p =* 0.001; Figure 4). However, *S. japonicum* adults had no significant preference between *B. tabaci*-infested cucumber and tobacco (t_6_ = −1.034, *p =* 0.341) or between *B. tabaci*-infested cotton and tobacco (t_6_ = −0.827, *p =* 0.440), although the numbers of ladybeetles appeared to have risen on *B. tabaci*-infested cucumber or cotton (Figure 4).

### 3.3. Y-Tube Olfactometer Experiments

**Figure 5:**
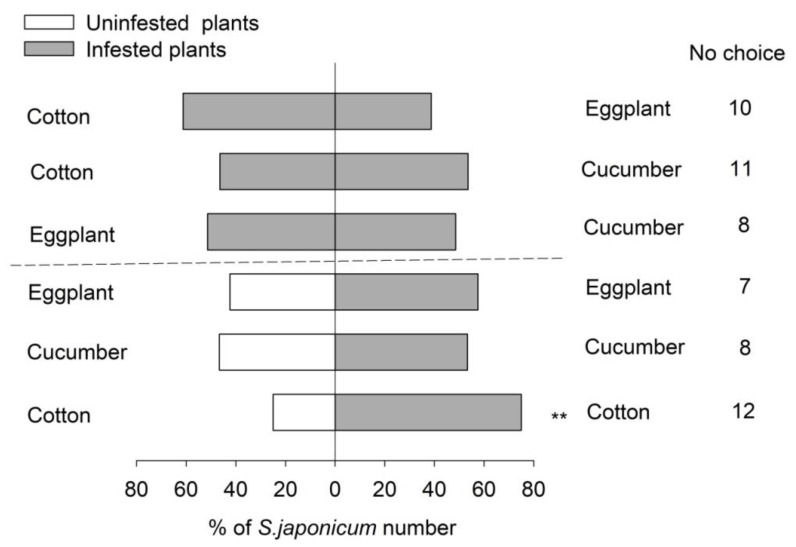
Two-choice assays of *Serangium japonicum* by Y-tube olfactometer. No choice means that the ladybeetles did not respond in the allotted time. Significance was analyzed in each group at *p <* 0.05. ** Represents values that were statistically significant at 0.01 > *p >* 0.001.

*Serangium japonicum* adults were able to recognize the odors emitted by *B. tabaci*-infested plants compared with uninfested plants in different plant species (Figure 5). More *S. japonicum* adults were attracted by *B. tabaci*-infested cotton compared with uninfested cotton (χ^2^ = 4.5, *p =* 0.043; Figure 5). *Serangium japonicum* adults had no obvious preference between *B. tabaci*-infested and uninfested eggplant (χ^2^ = 1.059, *p =* 0.303; Figure 5) or between *B. tabaci*-infested and uninfested cucumber (χ^2^ = 0.133, *p =* 0.715; Figure 5).

In addition, *S. japonicum* adults did not show an obvious response to volatiles from *B. tabaci-*infested cotton when presented against *B. tabaci-*infested eggplant (χ^2^ = 2, *p =* 0.157) and *B. tabaci-*infested cucumber (χ^2^ = 0.273, *p =* 0.602). When *B. tabaci-*infested eggplant and cucumber were presented, *S. japonicum* adults responded equally to both odors (χ^2^ = 0, *p* > 0.05; Figure 5).

### 3.4. Volatile Organic Compounds

**Figure 6:**
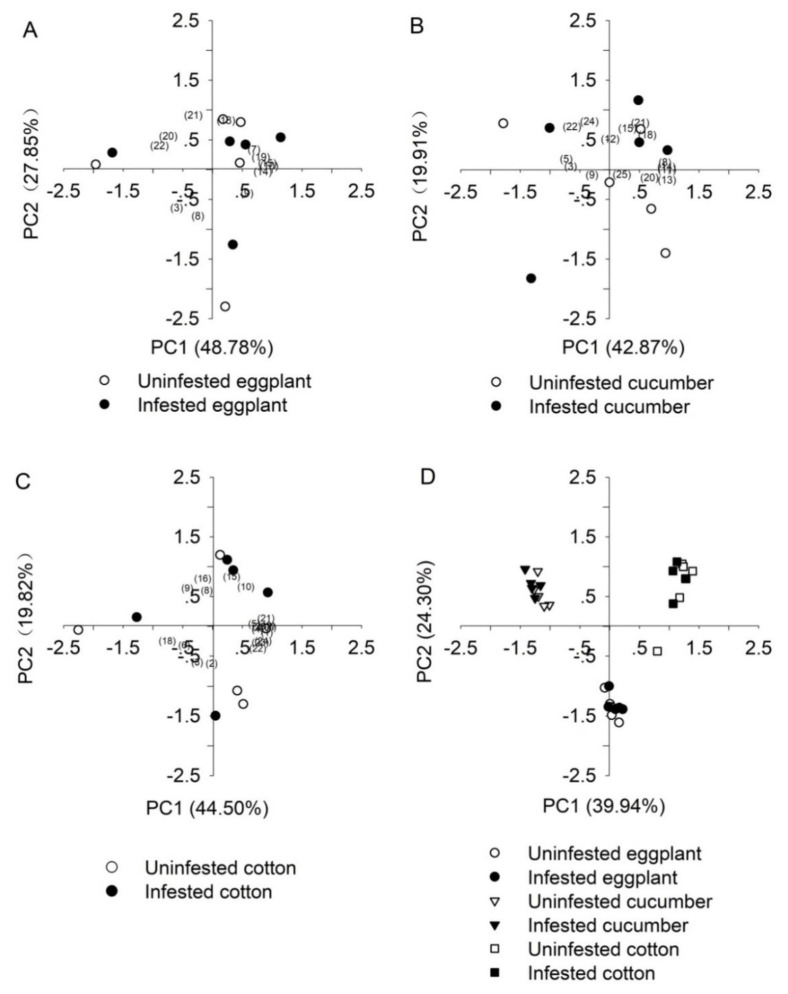
Principal component analysis (PCA) of volatile compounds from uninfested and *Bemisia tabaci*-infested host plant species. PCA with a factor loading plot was used to analyze single plant species, including eggplant (**A**), cucumber (**B**) and cotton (**C**). (**D**) PCA for three plant species, namely eggplant (circles), cucumber (triangles) and cotton (diamonds) based on the 25 identified volatile components. Score plots of PC1 and PC2 with the percentage of total variance explained by each axis are shown. Each point represents an individual plant. Compound numbers in Figure 6 correspond with compound numbers in Table 1.

No significant change was found in volatile composition between *B. tabaci-*infested and uninfested eggplant (Pillai test: F_(2, 114)_ = 0.100, *p =* 0.905). The volatile Unknown 1 was only detected in eggplant (Table 1). PCA results indicated that the first and second components explained 48.78% and 28.75% of the total variability, respectively (Figure 6A). According to the component matrix in PCA, the most important compounds were 2,6,10-trimethyl-tetradecane, Tridecane and Pentadecane for PC1; however, 3-methyl pentadecane and Heptadecane were the main substances for PC2 (Figure 6A). These results are consistent with the change in the relative content of volatiles in Table 1. The volatiles of uninfested and *B. tabaci*-infested eggplants were not distinguished by the PCA (Figure 6A).

Fifteen volatiles were detected from uninfested and *B. tabaci*-infested cucumber (Table 1). The volatile composition did not vary between *B. tabaci*-infested and uninfested cucumber plants (Pillai test: F_(2, 136)_ = 0.770, *p =* 0.926; Figure 6). The first two components of the PCA explained 62.79% of the total variability. Three components were unidentified. Unknown 2, Unknown 3 and 7-methyl-pentadecane were present in the blend of uninfested or *B. tabaci-*infested cucumber plants, but they were not found in eggplant or cotton. Heptadecane and tert-Hexadecanethiol had remarkable effects in terms of explaining PC2, and 2-ethenyl-1,1-dimethyl-3-methylene-cyclohexane, 2,6,10-trimethyl-tetradecane and 7-methyl-pentadecane played more important roles in explaining PC1 (Figure 6B). The volatiles of uninfested and *B. tabaci*-infested cucumber were not distinguished by the PCA (Figure 6B).

Cotton emitted more volatile species compared with those emitted by eggplant and cucumber plants (Table 1). There was no significant difference in volatile composition between *B. tabaci*-infested and uninfested cotton (Pillai test: F_(2, 183)_ = 0.620, *p =* 0.539, Figure 6C). The first two components of the PCA explained 64.32% of the total variability. β-Myrcene,1-Caryophyllene, Heptadecane and Humulene had remarkable effects in terms of explaining the first components of the PCA, and Pentadecane and Benzothiazole played a more important role in explaining the second components of the PCA (Figure 6C). The volatiles of uninfested and *B. tabaci*-infested cotton failed to be distinguished by the PCA (Figure 6C).

PCA showed the apparent separation of plant volatiles among eggplant, cucumber and cotton, but obvious overlaps were found in uninfested and *B. tabaci*-infested plants of the same species (Figure 6D). The first and second PCs explained 39.94% and 24.3% of the total variance of the plant volatiles, respectively. Not all compounds could be detected in different plant species; only nine common compounds were detected in all groups in this experiment (Table 1). 

## 4. Discussion

Most previous studies in this area have focused on the fact that plant species significantly affect the development, survival, population establishment and control efficiency of natural enemies [25,26,27], and a few works in the literature have focused on the behavior of natural enemies [28,29]. The ladybeetle *S. japonicum* has a great potential to suppress whitefly populations in an agricultural ecosystem. However, the locational behavior of *S. japonicum* toward *B. tabaci-*infested plants has seldom been reported. In the present study, we found support for our hypotheses that (1) *B. tabaci* adults are able to identify odors from different plants, and that *S. japonicum* adults can perceive volatile odors from different host plants of *B. tabaci*; and (2) uninfested and *B. tabaci*-infested eggplant are significantly preferred by *B. tabaci* and *S. japonicum*.

In the greenhouse experiments, *B. tabaci* and *S. japonicum* adults have clear preferences for different host plant species. *Bemisia tabaci* adults significantly preferred eggplant, cucumber and tobacco to cotton and tomato, and *S. japonicum* adults—a natural enemy of *B. tabaci*—preferred eggplant, cucumber, cotton, tobacco and tomato. Similar to our results, previous findings showed that eggplant was the most suitable host plant for *B. tabaci* and that tomato was the least suitable [30]. In addition, the survivorship from egg to adult of *B. tabaci* was highest on eggplant [31]. Eggplant was a suitable trapping plant to manage *Trialeurodes vaporariorum* (Westwood) (Hemiptera: Aleyrodidae) and *Bemisia argentifolii* Bellows & Perring (Hemiptera: Aleyrodidae) in poinsettia [32]. There are no earlier reports on the influence of the prey’s host plant species on the performance of *S. japonicum*, but cucumber was reported to be the most preferred plant by its sibling species *Serangium parcesetosum* Sicard (Coleoptera: Coccinellidae) compared with tomato [33]. Similarly, the predatory ladybeetle *Delphastus catalinae* (Horn) (Coleoptera: Coccinellidae) exhibited lower performance on *B. tabaci*-infested tomato [34]. *Serangium japonicum* laid more eggs on *B. tabaci*-infested eggplant and cucumber plants [35]. Interestingly, we found that uninfested tobacco plants could attract *B. tabaci* adults, but *B. tabaci*-infested tobacco plants were largely rejected by *S. japonicum* adults. The preference of *B. tabaci* for tobacco might be related to the high contents of 2-Hexenal and 3-Hexen-1-ol in tobacco [36]. Our previous data revealed that *B. tabaci*-infested tobacco was not an appropriate host for the development and oviposition of *S. japonicum* [35], but the reason that tobacco was rejected by *S. japonicum* was still unclear. These findings suggest that the host species of prey strongly affect the performance and fitness of predators. 

In the wind tunnel experiments, eggplant plants were highly preferred by *B. tabaci* adults compared with tomato plants, and *S. japonicum* adults preferred eggplant to tobacco. The results are consistent with the findings of our greenhouse experiments. In the Y-tube olfactometer experiment, *S. japonicum* could recognize uninfested cotton and *B. tabaci*-infested cotton, but it did not successfully distinguish the odors from eggplant or cucumber plants. Furthermore, egg deposition by *B. tabaci* did not make eggplant and cucumber more attractive to *S. japonicum* adults than uninfested eggplant and cucumber. Therefore, it is suspected that *S. japonicum* may need other factors (vision or taste sense) to locate its prey’s host plants. *Harmonia axyridis* depended on its smell sense and vision when looking for prey [37]. *Propylaea japonica* was reported to utilize olfactory and visual cues to locate aphid-infested cotton but mainly relied on olfactory odors [7]. At the same time, an earlier article reported that the attraction behavior of *H. axyridis* toward *Myzus persicae* (Sülzer) (Hemiptera: Aphididae) infested cabbage was affected by aphid density [21]. Thus, another explanation for our results is that different host plant species process different leaf areas, and the same density of *B. tabaci* adults possibly results in different damage intensities. Finally, *S. japonicum* adults failed to identify the odors from host plants in Y-tube bioassays. In addition, the orientational behavior of *S. japonicum* may not only need a combination of vision and olfactory organs but also a certain learning behavior. Ponsonby and Copland (1995) reported that experienced *Chilocorus nigritus* (F.) (Coleoptera: Coccinellidae) adults were attracted by prey and the prey’s host plants [38]. Naïve predatory mirid bug *Dicyphus hesperus* Knight (Hemiptera: Miridae) had no preference between uninfested and *Bactericera cockerelli* Sulcer (Hemiptera: Triozidae) egg-infested tomato; however, *D. hesperus* females with foraging experience preferred infested tomato [39]. In addition, the morphological characteristics of plants, such as trichome, leaf color, leaf shape and so on, also affect the seeking behavior of pests and natural enemies [40,41].

Our results showed that the different host plants emitted different kinds of volatile chemicals. The constituents of volatiles were different among three plant species, and they qualitatively varied between uninfested and *B. tabaci*-infested plants of the same species. Cotton released the most volatile species, while eggplant emitted the least volatile species. A total of nine substances were detected in three plant species simultaneously, and it is speculated that these substances might play an important role in the host-seeking behavior of *S. japonicum*. Nonanal and (Z)-3-hexenyl acetate were detected in *Spodoptera litura* Fabricius (Lepidoptera: Noctuidae)-infested eggplant [42]. The volatile Nonanal from *Sophora japonica* flowers markedly attracted *H. axyridis* [43], and a high content of Nonanal was also found in plant odors that attracted *Frankliniella occidentalis* (Pergande) (Thysanoptera: Thripidae) [40]. Many alkane species were detected and even reported in other plants [43,44]. Furthermore, the present results found that four volatiles, D-limonene, β-ocimene, Caryophyllene and Benzaldehyde, were identified in plant species preferred by *S. japonicum*. We speculate that these four substances might play an important role in the host-seeking behavior of *S. japonicum*. D-Limonene and β-ocimene contents were significantly correlated with the olfactory response of *P. japonica* [8]; Caryophyllene and Benzaldehyde attracted predatory lacewings [45,46]. Further studies are needed to explain the function of these substances in the orientational behavior of *S. japonicum*. In addition, PCA results showed that different plant species could be distinguished by the content of volatiles, but the content of volatiles failed to separate the same plant species in uninfested and *B. tabaci*-infested conditions. However, according to the Y-tube olfactometer assays, between *B. tabaci*-infested and uninfested plants, *S. japonicum* distinguished the odor from cotton only and did not recognize the odors from eggplant or cucumber. When a pairwise combination of three different *B. tabaci-*infested hosts acted as odors, the orientational choice of *S. japonicum* showed no significance. Thus, it is suspected that other factors (such as vision sense)—not only the smell sense—play a role in the localization behavior of *S. japonicum*.

An “attract and reward” model can use non-target plants grown within or around the target crop to alleviate pest populations, and it also can attract more natural enemies to the crop [41,47]. Our results showed that eggplant was the favorite host plant of *B. tabaci* and the predatory ladybeetle *S. japonicum*. Thus, we propose an innovative method—i.e., an eggplant + *B. tabaci* + *S. japonicum* system—to control *B. tabaci* in greenhouses and fields. In this system, eggplant can act as a trap plant to attract *B. tabaci* and reduce its damage to target plants before the rapid increase in the *S. japonicum* population. Subsequently, with the increase in the number of *S. japonicum*, the ladybeetles actively migrate from eggplant to target crops and exert control over *B. tabaci* on target crops.

## 5. Conclusions

The present study investigated the influence of host plant species of *B. tabaci* on the orientational behavior of *S. japonicum.* Our findings suggest that the host plant species preferred by *B. tabaci* are not necessarily also preferred by its natural enemy *S. japonicum*. The eggplant plant is the most preferred host plant for *B. tabaci* and *S. japonicum*, and the ladybeetle can perceive volatile odors from different host plants of *B. tabaci*. Since its prey’s host plant species can affect the orientational performance of *S. japonicum*, plant species should be considered when designing biological control programs that utilize *S. japonicum* to control *B. tabaci* populations. Accordingly, from the present results, we propose a new pest control strategy—i.e., an eggplant + *B. tabaci* + *S. japonicum* system—to control *B. tabaci* injury in greenhouses and fields. Moreover, our results also provide insight into the plant–whitefly–ladybeetle tritrophic interactive relationship and demonstrate that the successful biological control of pests should integrate host plants into the control strategy. 

## Figures and Tables

**Table 1:** Relative contents (mean ± SE) of volatiles from uninfested and *B. tabaci*-infested plants that were preferred by *Serangium japonicum*.

N	Chemical Compounds	Relative Content (%)					
		Uninfested Eggplant	*B. tabaci*-Infested Eggplant	Uninfested Cucumber	*B. tabaci*-Infested Cucumber	Uninfested Cotton	*B. tabaci*-Infested Cotton
(1)	β-Myrcene	-	-	-	-	1.17 ± 0.39	0.90 ± 0.33
(2)	1-Decyne	-	-	-	-	14.24 ± 3.07	15.82 ± 3.53
(3)	d-Limonene	5.59 ± 1.43	2.52 ± 1.07	9.39 ± 4.85	13.47 ± 8.98	3.03 ± 1.19	3.49 ± 1.25
(4)	(Z)-3-Hexenyl acetate	-	-	-	-	1.33 ± 0.33	1.56 ± 0.13
(5)	β-Ocimene	-	-	22.66 ± 14.06	11.09 ± 2.13	0.67 ± 0.21	1.17 ± 0.49
(6)	Benzaldehyde	-	-	-	-	8.98 ± 3.37	7.67 ± 2.53
(7)	Unknown1	5.77 ± 1.87	3.86 ± 1.54	-	-	-	-
(8)	2-Ethenyl-1,1-dimethyl-3-methylene-cyclohexane	11.55 ± 7.22	18.75 ± 9.62	4.94 ± 1.43	2.25 ± 0.69	23.83 ± 12.29	11.51 ± 5.17
(9)	Nonanal	14.30 ± 1.53	16.15 ± 2.62	5.51 ± 1.40	5.01 ± 1.80	4.06 ± 1.19	6.44 ± 1.70
(10)	Tridecane	1.45 ± 0.32	1.51 ± 0.46	-	-	1.00 ± 0.12	1.80 ± 0.52
(11)	Unknown 2	-	-	6.37 ± 1.54	5.69 ± 1.23	-	-
(12)	Unknown 3	-	-	1.22 ± 0.37	1.41 ± 0.38	-	-
(13)	7-Methyl-pentadecane	-	-	2.28 ± 0.63	1.75 ± 0.36	-	-
(14)	2,6,10-Trimethyl-tetradecane	1.95 ± 0.61	2.16 ± 0.39	1.33 ± 0.29	0.85 ± 0.29	3.11 ± 0.62	5.13 ± 1.10
(15)	Pentadecane	3.75 ± 0.64	4.47 ± 1.00	4.16 ± 0.64	3.47 ± 0.61	2.18 ± 0.27	3.93 ± 0.62
(16)	Benzothiazole	-	-	-	-	0.65 ± 0.16	1.27 ± 0.30
(17)	β-Caryophyllene	-	-	-	-	15.33 ± 9.75	3.53 ± 1.29
(18)	3-Methyl-pentadecane	1.11 ± 0.29	1.13 ± 0.25	1.03 ± 0.20	0.99 ± 0.44	2.61 ± 2.08	5.79 ± 3.23
(19)	Humulene	2.91 ± 0.84	2.71 ± 0.52	-	-	4.27 ± 2.80	0.96 ± 0.29
(20)	2,6,10-Trimethyl-pentadecane	10.02 ± 1.21	10.00 ± 1.76	14.95 ± 1.14	16.32 ± 2.67	3.90 ± 1.45	7.91 ± 1.64
(21)	Heptadecane	14.89 ± 2.54	15.70 ± 2.57	13.30 ± 2.69	16.87 ± 2.89	5.59 ± 0.79	9.65 ± 2.05
(22)	2,6,10,14-Tetramethyl hexadecane	16.67 ± 5.76	12.72 ± 4.99	15.56 ± 2.07	21.69 ± 2.41	6.89 ± 2.94	11.15 ± 3.45
(23)	á-Bisabolol	-	-	-	-	2.04 ± 0.88	0.68 ± 0.24
(24)	tert-Hexadecanethiol	-	-	2.23 ± 0.39	2.74 ± 1.85	1.51 ± 0.75	1.35 ± 0.29
(25)	Unknown 4	-	-	1.27 ± 0.49	0.69 ± 0.07	-	-
